# A Survey on the Use of Outcome Measures during Physical Therapy Interventions by Physical Therapists in Korea

**DOI:** 10.3390/healthcare11222933

**Published:** 2023-11-09

**Authors:** Jae-Hyun Lim, So-Yeong Kim, Byeong-Geun Kim

**Affiliations:** 1 Department of Physical Therapy, Graduate School, Nambu University, Gwangju 62271, Republic of Korea; jhjhoss@naver.com (J.-H.L.); belleyou11@naver.com (S.-Y.K.); 2 Physical Therapy Room, Wise Rehabilitation Hospital, Gwangju 62421, Republic of Korea; 3 Rehabilitation Center, Gwangju 365 Rehabilitation Hospital, Gwangju 62232, Republic of Korea; 4 Department of Physical Therapy, Nambu University, Gwangju 62271, Republic of Korea

**Keywords:** physical therapy, physical therapist, outcome measures, survey

## Abstract

The purpose of this study was to determine the current status of patient care provided by Korean physical therapists (KPTs) in clinical practice by studying the outcome measures (OMs) used in physical therapy interventions among KPTs with experience in treating patients. A total of 225 KPTs with experience in treating patients in clinical settings participated in the study and completed the online questionnaire. The questionnaire included questions about the use of OMs and the reasons for using them, as well as the types, benefits, and barriers of OMs. The participants’ responses were analyzed and reported in terms of frequencies and percentages. A total of 220 questionnaires were analyzed. The results show that the majority of KPTs in clinical practice used OMs during interventions. The main reasons for using OMs were to check the patient’s condition and to determine the direction and effectiveness of treatment. In terms of the types of OMs used, the highest percentage of subjects used both patient-reported OMs (PROMs) and performance-based OMs (PBOMs). They chose OMs that were quick and easy to use and used them voluntarily. Barriers to and reasons for not using OMs were similar, including lack of benefits, lack of time, and problems with patient performance and uncooperative behavior. When analyzing the effect of demographic characteristics on the use of OMs, we found that physical therapists specializing in musculoskeletal and neurological systems, physical therapists with longer treatment times, and physical therapists who valued OMs were more likely to use them. Based on the results of this study, it is recommended that improvements in the work environment and healthcare system are needed to enhance the professionalism of KPTs working in the field of physical agent therapy by improving their awareness of Oms and improving the quality of physical therapy interventions.

## 1. Introduction

Outcome measures (Oms) are methods used to determine a patient’s current status [1] and are a component of evidence-based practice (EBP) that can provide physical therapists with the information they need to determine a patient’s status [2]. Oms have been used globally for decades in developed countries and are an integral part of clinical practice, with the importance of Oms being recognized by all healthcare professionals, including physical therapists [3]. And recent advances in healthcare have emphasized the importance of patient-centered care to reduce disability and improve quality of life [4]. In addition, the involvement of insurance companies as third-party payers has put pressure on physical therapists to demonstrate the effectiveness of treatment and has led to accountability requirements for all health professionals, including physical therapists, to assess patient satisfaction with treatment and the level of effectiveness of treatment [5,6,7]. These needs can be addressed by Oms that can monitor the patient’s disability, pain management, physical impairment, and health status while identifying changes in the patient’s condition [8,9].

The two types of OMs are patient-reported outcome measures (PROMs) and performance-based outcome measures (PBOMs). PROMs are defined as a patient’s own assessment of their health status, without input from a doctor or therapist [10]. PBOMs are one way of assessing a patient’s functional ability, either using a tool in the clinic or by the therapist themselves [11]. PROMs and PBOMs are used to quantify a patient’s functional ability [12]. OMs are used in clinical practice to inform patients, therapists, managers, and funders about the effectiveness and achievability of treatment goals and in research to compare the effects of two interventions in controlled trials to determine the effectiveness of one over the other [13]. In particular, physical therapists utilize OMs to enhance and support clinical decision making, determine treatment effectiveness, improve rehabilitation services, and motivate patients to improve their ability to perform activities of daily living [14]. They also compare the effectiveness of treatments with other healthcare professionals, keep patients informed about their progress, and plan effective courses of treatment [15]. As such, physical therapists benefit from using OMs in their interventions.

Inglis et al. [16] conducted a study on the awareness and use of OMs among physical therapists in South Africa and reported that few rehabilitation centers routinely use OMs in their practice. Alreni et al. [17] conducted a study on OMs in the intervention of nonspecific neck pain among physical therapists in the United Kingdom and reported that they used simple pain scales, range of motion scales, or no OMs when managing patients with nonspecific neck pain. Östhols et al. [18] reported a low to very low level of use of PROMs in their study on Swedish physical therapists in interventions with patients with back pain.

Although there have been previous studies conducted on OMs for foreign physical therapists, there is a lack of research conducted on OMs utilized by Korean physical therapists. Therefore, the purpose of this study was to determine the current status of patient care provided by physical therapists in clinical practice by conducting a study on the OMs used during physical therapy intervention among Korean physical therapists who have experience in patient intervention and to provide a basis for the upward development of physical therapy in Korea and suggest future directions for the field.

## 2. Methods

### 2.1. Study Procedure

This study was approved by the Institutional Review Board of Nambu University (1041478-2023-HR-004). The purpose of this study was to determine whether Korean physical therapists, who have experience in patient intervention in clinical practice, use OMs and to analyze their reasons for doing so, the types of OMs used, the benefits, and barriers of using OMs, and the effect of general characteristics on their use of OMs.

### 2.2. Participants

The selection criteria for this study included licensed physical therapists practicing in Korea with experience in physical therapy interventions. The exclusion criteria consisted of physical therapists without any experience in physical therapy interventions in clinical practice. The sample size for this study was determined using G*power 3.1.9.7 to allow for chi-squared analysis. With the highest degree of freedom being 7 among the survey items, an effect size of 0.3, a significance level of 0.05, and a power of 0.9, the calculated minimum sample size was 204 subjects. Considering a dropout rate of 10%, our aim was to recruit 225 subjects.

### 2.3. Development of the Survey

The questionnaire used in this study was developed appropriately for the Korean clinical setting by collecting OMs items through a systematic review [19] and conducting focus group interviews [20]. The interview involved four physical therapists with more than 10 years of clinical experience and four physical therapists with more than 3 years but less than 10 years of clinical experience. The purpose of the interview was to strengthen the construct and validity of the questionnaire and to reflect the opinions of the participants in order to apply the questionnaire to Korean physical therapists (Table 1). The questionnaire consisted of nine general characteristics, including gender, age, highest level of education, work experience, work setting, treatment time, working field, monthly salary, importance of OMs, whether they used OMs, reasons for using OMs, reasons for not using OMs, types of OMs, benefits of OMs, and barriers of OMs. Physical therapists who use OMs were asked to complete a survey about the reasons for using, types, benefits, and barriers of Oms. Physical therapists who do not use Oms were asked to complete a survey about their reasons for not using Oms.

### 2.4. Data Collection

The survey was conducted over five days using an online Google Form. Subjects were recruited through online community sites and social media platforms commonly used by most physical therapists in Korea. The consent form prior to the start of the survey informed participants that participation was voluntary, that they could withdraw from the survey at any time, that there would be no penalty for withdrawal, and that their confidentiality would be protected. The purpose of the study was explained, consent to participate in the study and use of personal information was obtained, and they were asked to provide their license number to verify that they were a physical therapist. Participants who agreed to take part in the survey were able to do so via the QR code or survey address link provided in the recruitment announcement, and those who participated voluntarily took the survey, which took approximately 10 min to complete.

### 2.5. Data Analysis

Data from this study were analyzed using IBM SPSS software version 25.0. The responses to the general characteristics of the participants, whether they used OMs and their reasons for using it, and the types, benefits, and barriers of OMs were expressed as frequencies and percentages. Chi-square test and Fisher’s exact test were used to test for differences in the presence of OMs according to general characteristics, and logistic regression analysis was used to analyze the effect of significant general characteristics on the presence of OMs. The level of statistical significance was set at 0.05.

## 3. Results

### 3.1. Participants

A total of 225 questionnaires were collected through the online survey, and after excluding 5 questionnaires with duplicate answers, 220 questionnaires were finally collected. A total of 53.2% of participants were male, and the most common age was 26–30 years (48.6%). The highest percentage of participants had a bachelor’s degree (58.2%), and the highest percentage of physical therapists had five or fewer years of experience (54.1%). The largest number of physical therapists (104) worked in clinics and Korean medicine clinics (47.3%), with the majority of physical therapists working with the musculoskeletal system (55.5%) and the nervous system (34.5%). A large proportion (37.7%) treated clients for more than 15 min and less than 30 min. Most therapists were earning between KRW 2 and KRW 2.99 million per month (55.5%) or more than KRW 3 million per month (39.1%). Regarding the importance of OMs, the majority of physical therapists considered OMs to be very important (73.2%) or somewhat important (25.5%), and no physical therapists considered OMs to be negative. Chi-squared analysis revealed significant differences in gender (*p* < 0.001), area of practice (*p* < 0.000), treatment time (*p* < 0.015), monthly salary (*p* < 0.019), and the importance of OMs (*p* < 0.000) (Table 2).

### 3.2. Whether They Use OMs

A total of 84.1% of physical therapists reported they used OMs often (43.2%) and always (40.9%), and 15.9% of physical therapists rarely (12.3%) or never (3.6%) used OMs (Table 3).

### 3.3. Reasons for Using Outcome Measures

The main reasons why participants used OMs were to check patient status (4.78 ± 0.43), determine treatment direction (4.72 ± 0.48), identify changes in patient status (4.7 ± 0.47), improve quality of care (4.46 ± 0.65), identify and document patient status, progress, and treatment effects (4.02 ± 0.97), compare treatment effects between patients (3.62 ± 1.2), communicate with other healthcare providers (3.16 ± 1.18), compare treatment effects between therapists (3.1 ± 1.46), and for research (2.13 ± 1.12).

### 3.4. Type of Outcome Measures

Altogether, 106 (57.3%) physical therapists used both PROMs and PBOMs, 59 (31.9%) physical therapists used only PBOMs, and 20 (10.8%) physical therapists used only PROMs. The conditions under which OMs were used were as follows: voluntary depending on the patient’s condition or environment (28.1%) or voluntary for all types of patients (22.7%). A total of 50.8% of participants used OMs voluntarily (Table 4). The main reasons for choosing OMs were that they were easy for therapists to use (64.3%) and quick to complete (60.5%). More than half also chose those that were widely used in clinical practice by physical therapists (54.1%) and easy for patients to understand (50.8%).

### 3.5. Benefits of Outcome Measures

The main benefits of using OMs listed by the participants were help with treatment planning (4.58 ± 0.53), help with treatment choice (4.54 ± 0.63), increased treatment efficiency (4.44 ± 0.62), help with therapists and patients communication (4.43 ± 0.69), better patient outcomes (4.41 ± 0.66), help with motivating and encouraging patients (4.39 ± 0.69), making patients feel that the therapists are thorough (4.36 ± 0.73), help in communicating with third-party payers and other healthcare providers (3.87 ± 0.9), and help with marketing the clinic/service (3.85 ± 0.99).

### 3.6. Barriers to Using Outcome Measures

The main barriers to OMs listed by the participants were that they are ineffective (3.57 ± 1.29), are difficult for patients to perform themselves (3.46 ± 0.95), require more effort than patients are capable of (3.42 ± 0. 94), have an insufficient treatment time (3.38 ± 1.02), are time-consuming (3.2 ± 0.97), can be hindered by an uncooperative patient attitude (3.01 ± 1.01), are time-consuming to analyze/calculate/evaluate (3 ± 0.98), and led to results that are difficult to trust (2.75 ± 0.88).

### 3.7. Reasons for Not Using Outcome Measures

**Figure 1:**
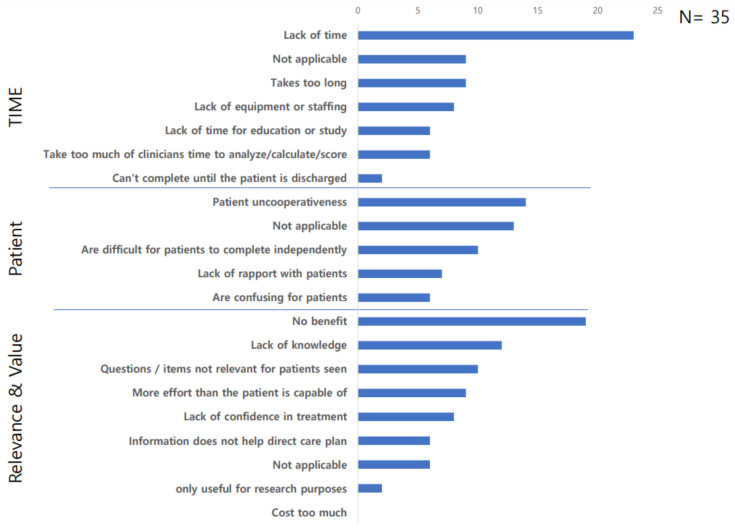
Reasons for not using outcome measures.

The main reasons participants cited for not using OMs were lack of time (65.7%), lack of benefits (54.3%), and patient uncooperativeness (40%) (Figure 1).

### 3.8. Reasons for Not Using Outcome Measures (Other)

Other reasons given for not using OMs were that most of their patients are chronic and would rather be treated than use OMs, they do not want the hospital to use OMs, because it is a routine task, and because it is their time to treat patients.

### 3.9. Effect of Participants’ General Characteristics on Whether They Use OMs

Logistic regression was used to analyze the effect of gender, working field, monthly salary, and perceived importance of OMs on the use of OMs, as these were the most common characteristics that showed significant differences. Other as a working field was excluded from the analysis due to the low frequency of physical therapists not using OMs. The logistic regression model was statistically significant (Hosmer & Lemeshow x^2^ = 6.788, *p* = 0.560), and the explanatory power of the regression model was approximately 35.2% (Nagelkerke R^2^ = 0.352). The results of the analysis indicate that the musculoskeletal system (OR = 15.5, *p* < 0.01), the neurological system (OR = 9.3, *p* < 0.01), treatment times exceeding 45 min (OR = 12.0, *p* < 0.01), treatment times ranging from more than 30 min to less than 45 min (OR = 4.9, *p* < 0.05), and the perceived importance of OMs (OR = 5.2, *p* < 0.001) significantly influenced whether physical therapists used OMs (Table 5).

## 4. Discussion

In this study, we conducted a survey of Korean physical therapists with experience in patient intervention to determine the actual use of OMs and factors influencing the use of OMs and to provide basic data and suggest future directions for the upward development of Korean physical therapy.

Regarding the types of patients for whom they use OMs, half of the physical therapists reported using OMs voluntarily for all types of patients or certain types of patients, and one third reported using OMs mandatorily for all types of patients or certain types of patients. In a study similar to this one, physical therapists in India also reported mandatory use of OMs in the workplace [3]. A previous study on Saudi Arabian physical therapists also reported that half of the participants were required to use OMs at work, and that high organizational commitment and support at work can promote the use of OMs [21]. Therefore, there is a need to improve the work environment to encourage physical therapists in Korea to use OMs in patient interventions. In terms of the types of OMs used by Korean physical therapists, the highest proportion used both PROMs and PBOMs. It was reported that PROMs and PBOMs have a moderate correlation and that the two types of OMs complement each other because they assess different scopes [22]. Therefore, it is necessary to use PROMs and PBOMs appropriately, as is practiced by the majority of physical therapists in Korea.

Korean physical therapists’ barriers to using OMs and reasons for not using OMs were similar, with common themes including a lack of benefits and lack of treatment time. Physical therapists in India reported that they did not use OMs because there were no mandatory or legal requirements and no incentives to use OMs [3]. This is also the case in South Korea, where a previous study reported that fee-for-service clinicians used OMs more than salaried clinicians [23]. Therefore, the Korean physical therapy system should be improved, and the use of OMs should be encouraged and made mandatory to increase the income of physical therapists and improve the quality and service of physical therapy interventions. Lack of treatment time was identified in many previous studies [3,14,17,21] and is consistent with the findings of this study. Physical therapists in India reported difficulties using OMs because they treat 10 to 12 patients every 8 h [3], and physical therapists in Colombia reported limitations in practicing EBP because they treat 11 patients per day for 31 h per week [24]. It is assumed that the clinical environment of Korean physical therapists is also similar, which may have contributed to their difficulties in using OMs.

In this study, gender, age, monthly salary, highest level of education, and clinical experience did not have a significant effect on the use of OMs by Korean physical therapists. Gender differences in the use of OMs were not significant. Previous research has also reported no gender differences in EBP practice among healthcare professionals [25]. However, a contrary finding that men had higher self-efficacy in practicing EBP than women among physical therapists has been reported, and this was attributed to several factors, including higher education, social status, and income [26]. Therefore, the influence of gender on the use of OMs also warrants further research. In addition, previous studies have also reported no significant differences in attitudes toward EBP based on age, clinical experience, and education, which is consistent with this study [3]. These results suggest that the current curriculum does not influence the use of OMs. EBP curricula were reported to be effective in improving attitudes and skills [27], and familiarity with the research process without a master’s degree was reported to increase the use of OMs [28]. In Korea, it is necessary to improve the core curriculum and provide research-related education to enable physical therapists to use OMs in clinical practice.

In this study, the majority of Korean physical therapists considered it important to use OMs, but less than half of the participants reported always using them in their clinical practice. Dutch physical therapists reported positive attitudes towards the use of OMs and were aware of the benefits of using measurement tools but were unable to use them in their daily practice [29]. A systematic review found that although the importance of OMs is recognized, they are little used in practice, and the main barriers include issues related to clinician knowledge, low organizational priority and support, lack of time, lack of appropriate or available OMs, and OMs that do not support practice [15]. Therefore, the use of OMs in Korean clinical settings will require efforts by physical therapists and policy changes to address the main barriers.

A limitation of this study was the lack of introduction of the tools used by Korean clinical physical therapists to conduct OMs. Physical therapists in India and the Netherlands mostly used OMs but also reported using the VAS (Visual Analog Scale), MMT (Manual Muscle Test), and ROM (Range of Motion) scales [3,17]. These simple tools did not provide specific information about the patients, such as physical functioning, psychological factors, social competence, or quality of life [30]. Participants in this study also reported choosing tools that were easy and quick to use, and it is likely that they used one-dimensional scales. Therefore, it is recommended that follow-up studies introduce various tools for the use of OMs and conduct realistic and practical surveys for reference and use.

## 5. Conclusions

The majority of Korean physical therapists use OMs when intervening with patients to improve the quality of care and monitor their condition. OMs are an essential component of EBP, and Korean physical therapists are found to practice them. The main factors associated with the use of OMs were physical therapists working in physical agent therapy, physical therapists with insufficient treatment time, and physical therapists who did not consider it important to use OMs. It is suggested that efforts by physical therapists and policy changes are needed to address barriers to the use of OMs. Further research should investigate the types of OMs tools that Korean physical therapists use when intervening with patients in clinical practice.

## Figures and Tables

**Table 1:** Development of the survey.

Steps	Method	Content	Study Subjects
Step 1	Systematic review	Collection of OM survey questions	-
Step 2	Focus group interviews	Conduct focus group interviews to improve the construction and validity of the survey questions collected in step 1	Physical therapist with at least 3 years of clinical experience
Step 3	Survey	Collect and analyze OM data using the questionnaire constructed in steps 1 and 2	Physical therapists with clinical experience in physical therapy interventions

**Table 2:** General characteristics.

Characteristics	Category	Whether They Use OMs		Total (%)	x^2^	*p*
		Yes (%)	No (%)			
Gender	Male	107 (57.8)	10 (28.6)	117 (53.2)	10.125 **	0.001
	Female	78 (42.2)	25 (71.4)	103 (46.8)		
Age	20–25	24 (13)	10 (28.6)	34 (15.5)	6.631	0.085
	26~30	90 (48.6)	17 (48.6)	107 (48.6)		
	31~35	42 (22.7)	5 (14.3)	47 (21.4)		
	36~	29 (15.7)	3 (8.6)	32 (14.5)		
Highest level of education	Associate degree	49 (26.5)	14 (40)	63 (28.6)	3.746	0.154
	Bachelor	109 (58.9)	19 (54.3)	128 (58.2)		
	Master’s or higher	27 (14.6)	2 (5.7)	29 (13.2)		
Work experience	1~5 yrs	98 (53)	21 (60)	119 (54.1)	2.393	0.302
	6~10 yrs	51 (27.6)	11 (31.4)	62 (28.2)		
	11 yrs~	36 (19.5)	3 (8.6)	39 (17.7)		
Work setting	Clinic/Korean medicine clinic	85 (45.9)	19 (54.3)	104 (47.3)		0.158 ^†^
	General Hospital or higher	27 (14.6)	3 (8.6)	30 (13.6)		
	Convalescent/rehabilitation hospitals	47 (25.4)	12 (34.3)	59 (26.8)		
	Others ^a^	26 (14.1)	1 (2.9)	27 (12.3)		
Working field	Musculoskeletal	110 (59.5)	12 (34.3)	122 (55.5)		0.000 ***^,†^
	Nervous	62 (33.5)	14 (40)	76 (34.5)		
	Physical agent therapy	5 (2.7)	9 (25.7)	14 (6.4)		
	Others ^b^	8 (4.3)	0 (0)	8 (3.6)		
Treatment time (min)	~14	14 (7.6)	7 (20)	21 (9.5)	10.526	0.015 *
	15~29	66 (35.7)	17 (48.6)	83 (37.7)		
	30~44	57 (30.8)	8 (22.9)	65 (29.5)		
	45~	48 (25.9)	3 (8.6)	51 (23.2)		
Monthly salary (million, in KRW)	~1.99	11 (5.9)	1 (2.9)	12 (5.5)	7.926	0.019 *
	2~2.99	95 (51.4)	27 (77.1)	122 (55.5)		
	3~	79 (42.7)	7 (20)	86 (39.1)		
Importance of OMs	Very important	145 (78.4)	16 (45.7)	161 (73.2)		0.000 ***^,†^
	Somewhat important	39 (21.1)	17 (48.6)	56 (25.5)		
	Moderately important	1 (0.5)	2 (5.7)	3 (1.4)		
	Not very important	0	0	0		
	Not at all important	0	0	0		
Total		185	35	220		

Others ^a^: national hospitals, public hospitals, public corporations, health centers, social welfare organizations, sports centers; others ^b^: elderly, cardiopulmonary, hydrotherapy, sports, children. ^†^ Fisher’s exact test; * *p* < 0.05, ** *p* < 0.01, *** *p* < 0.001.

**Table 3:** Whether they use OMs.

		N	%
Whether they use OMs	Always	90	40.9
	Often	95	43.2
	Rarely	27	12.3
	Never	8	3.6

**Table 4:** Types of OMs.

	N	(%)
Types of OMs used		
Use both	106	57.3
Performance-based outcome measures	59	31.9
Patient-report outcome measures	20	10.8
Conditions under which OMs were used		
Voluntary OMs based on patient condition or environment	52	28.1
Voluntary OMs for all types of patients	42	22.7
Mandatory OMs based on patient condition or environment	33	17.8
Mandatory OMs for all types of patients	33	17.8
Depending on various factors such as time, patient characteristics, etc.	25	13.6

**Table 5:** The effect of general characteristics on the use of OMs.

		B	S.E	OR	95% CI	*p*
Gender	Female vs. Male	0.2	0.5	1.2	(0.4~3.6)	0.623
Working field	Physical agent therapy vs. Musculoskeletal	2.7	0.8	15.5 **	(3.2~75.2)	0.001
	Physical agent therapy vs. Neurological	2.2	0.7	9.3 **	(2.1~40.3)	0.003
Treatment time(min)	~14 vs. 15~29	1.1	0.7	3.0	(0.7~12.6)	0.117
	~14 vs. 30~44	1.5	0.7	4.9 *	(1.1~20.2)	0.028
	~14 vs. 45~	2.4	0.9	12.0 **	(1.8~78.0)	0.009
Monthly salary (million, in KRW)	~200 vs. 200~299	−0.9	1.1	0.4	(0.0~3.7)	0.427
	~200 vs. 300~	−0.9	1.2	0.3	(0.0~4.5)	0.451
	Importance of OMs	1.6	0.4	5.2 ***	(2.2~12.5)	0.000
−2LL = 142.172, NagelKerke R^2^ = 0.352, Hosmer & Lemeshow test: x^2^ = 6.788 (*p* = 0.560)						

Dummy variables reference categories: working field (physical agent therapy), treatment time (~15 min), monthly salary (KRW ~2 million), gender (female); * *p* < 0.05, ** *p* < 0.01, *** *p* < 0.001.

## Data Availability

Data can be requested from the corresponding author and will be released on reasonable request.
